# Evaluating Generative AI’s Ability to Identify Cancer Subtypes in Publicly Available Structured Genetic Datasets

**DOI:** 10.3390/jpm14101022

**Published:** 2024-09-25

**Authors:** Ethan Hillis, Kriti Bhattarai, Zachary Abrams

**Affiliations:** 1 Institute for Informatics, Data Science and Biostatistics, Washington University School of Medicine in St. Louis, St. Louis, MO 63110, USA; kriti.bhattarai@wustl.edu (K.B.); abramsz@wustl.edu (Z.A.); 2 Department of Computer Science, Washington University in St. Louis, St. Louis, MO 63130, USA

**Keywords:** large language models, gene expression, machine learning, predictive modeling, artificial intelligence

## Abstract

Background: Genetic data play a crucial role in diagnosing and treating various diseases, reflecting a growing imperative to integrate these data into clinical care. However, significant barriers such as the structure of electronic health records (EHRs), insurance costs for genetic testing, and the interpretability of genetic results impede this integration. Methods: This paper explores solutions to these challenges by combining recent technological advances with informatics and data science, focusing on the diagnostic potential of artificial intelligence (AI) in cancer research. AI has historically been applied in medical research with limited success, but recent developments have led to the emergence of large language models (LLMs). These transformer-based generative AI models, trained on vast datasets, offer significant potential for genetic and genomic analyses. However, their effectiveness is constrained by their training on predominantly human-written text rather than comprehensive, structured genetic datasets. Results: This study reevaluates the capabilities of LLMs, specifically GPT models, in performing supervised prediction tasks using structured gene expression data. By comparing GPT models with traditional machine learning approaches, we assess their effectiveness in predicting cancer subtypes, demonstrating the potential of AI models to analyze real-world genetic data for generating real-world evidence.

## 1. Introduction

Genetic data are important indicators and measures of human health. There are a multitude of diseases and conditions for which genetic testing is a vital and integral part of the diagnosis and treatment course [1]. As we learn more about the relationships of genotypes to phenotypes across different diseases and conditions, the importance of understanding genetic data grows, and the pressure to better leverage these data and information correspondingly increases. Currently, there are many roadblocks to the full integration of genetic data into clinical care [2]. Some of these include the structure of EHRs, insurance pricing on genetic testing, and the interpretability of genetic results. The present paper addresses some of these issues by leveraging a combination of new technological advancements with informatics and data science theory while employing novel experimental design to demonstrate the diagnostic value of AI in cancer research.

Artificial intelligence (AI) is a powerful tool in many different research fields. Historically, early versions of AI were applied in medical and biomedical research, but with limited success [3]. Recently, new technological developments have elevated a new form of AI referred to as large language models (LLMs) [4]. LLMs differ from previous iterations due to the increased volume of data they are trained on and the scale of their generative capabilities. These transformer-based generative AI models have great promise in furthering genetic and genomic analyses. They have been used for tasks such as candidate gene prioritization and selection, the text mining of biological pathways, the entity extraction of genetic interactions from unstructured data, etc. [5,6,7,8]. Nonetheless, there are important methodological limitations that we must first overcome [9,10]. Transformer models are highly dependent on the corpus of data they are trained on, and it seems those data are primarily confined to human-written forms of text data rather than large corpuses of publicly available genetic data [11].

Prior research has utilized traditional machine learning methods with gene expression data to predict cancer subtypes [12,13,14]. This paper evaluates the ability of LLMs, specifically GPT, to perform supervised prediction tasks leveraging only structured gene expression data. As aforementioned, large language models have been used for genetics; however, they have never been used in a setting such as this. We performed this evaluation using a set of GPT-based models and traditional machine learning models to evaluate the efficacy of AI models to analyze real-world data (gene expression) for real-world evidence (cancer subtype prediction) generation. 

## 2. Materials and Methods

**Figure 1:**
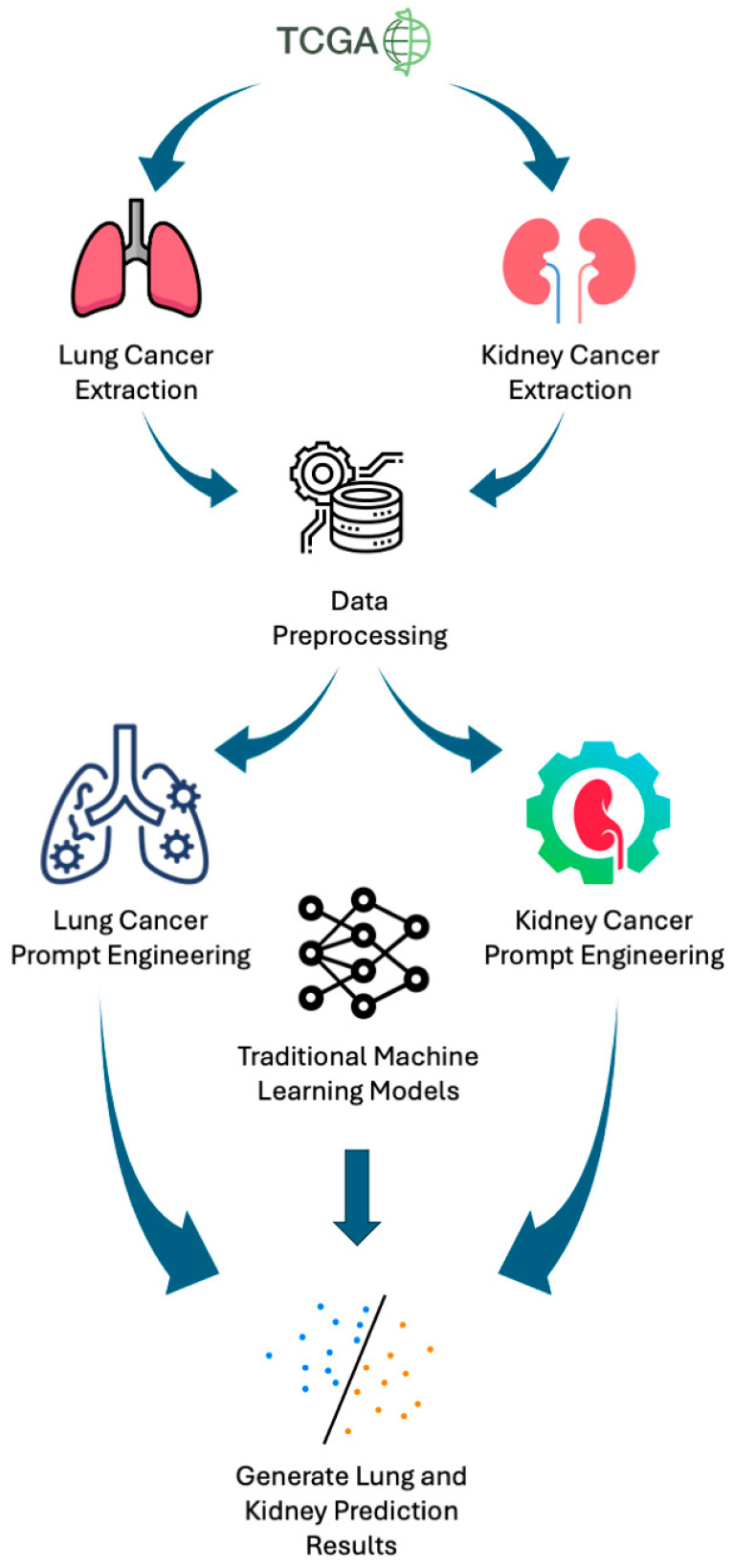
Experimental design workflow. Lung and kidney cancer datasets were extracted from TCGA and processed to perform feature reduction, keeping only the most informative genes. A total of 11 GPT prompts (7 for lung cancer prediction and 4 for kidney cancer prediction) were then created using different prompt engineering techniques. Lung and kidney cancer predictions are produced using the 11 GPT prompts and 5 traditional machine learning models and the results are evaluated.

An overview of our experimental design is shown in Figure 1.

Data Extraction

RNA-sequencing gene expression data were obtained through two different extractions from The Cancer Genome Atlas program (TCGA) available through https://www.cancer.gov/tcga accessed on 24 August 2023. The first set was gene expressions of lung cancer patients diagnosed with squamous cell carcinoma (SCC) or adenocarcinoma (AD) and gene expressions. This contained 1153 unique patients each with expression counts of 59,427 genes. The second set of extracted data was gene expressions of kidney cancer patients diagnosed with chromophobe renal cell cancer (ChRCC), clear cell renal cell cancer (CCRCC), or papillary renal cell cancer (PRCC). It contained 1029 unique patients each with expression counts of 59,427 genes.

Data Processing

Both lung cancer and kidney cancer data were preprocessed to reduce the high dimensionality of the data. To fit within GPT’s context window, a small subset of the ~59k genes were selected to maximize the data’s patterns and information structure. Principal component analysis (PCA) was applied to each dataset, with the number of components selected that aggregated to a 99% explained variance threshold. Next, the top 100 genes by absolute value in each component were aggregated, with the unique set of genes selected for the final dataset. For the lung cancer dataset, 116 components met the 99% explained variance threshold, and, after finding the unique set from the top 100 genes of each of the 116 components, there were 460 genes selected for analysis. For the kidney cancer dataset, the same workflow resulted in 58 components that yielded 431 unique genes selected for analysis. The complete lung cancer dataset contained 1153 patients and 460 genes, and the complete kidney cancer dataset contained 1029 patients and 431 genes.

The full datasets were then broken into training and testing sets. For each cancer type, a training and testing dataset was made for traditional machine learning algorithms and a training and testing dataset was made for GPT. For each cancer type, the training datasets contained the exact same samples/patients, their only difference being the formatting of the dataset. The same holds true for each testing dataset. The lung cancer training sets contained 40 samples, split evenly by SCC and AD labels, and the testing sets contained 1000 samples, again split evenly by label. The kidney cancer training sets contained 60 samples, split evenly by ChRCC, CCRCC, and PRCC, and the testing sets contained the 969 samples that were not in the training sets.

Prompt Engineering

Prompts are split up according to cancer dataset. While there was not a directed hypothesis for each prompt, the overall goal in creating different prompts was to provide the LLM with various forms of information to determine which resulted in the best performance. The true hypothesis was that providing information in engineered prompts would improve upon the baseline prompt. 

### 2.1. Lung Cancer Prompts (Supplementary Materials)

Basic: The Basic prompt asserts GPT’s role as a geneticist agent with the goal of diagnosing a lung cancer patient with SCC or AD. It then provides background information on the biology of what gene expression is and what it means for a gene to have high or low expression. Finally, it instructs GPT on the correct output format.

Feedback: The Feedback prompt is an aggregation of the Basic prompt with injected feedback on GPT’s previous prediction. After a GPT prediction, that prediction is checked for correctness and a one sentence feedback statement of what GPT’s prediction was and whether it was correct or incorrect is injected into the next sample’s prompt. This then repeats for every prediction such that the next feedback statement replaces the previous one. For example, if the GPT prediction of SCC for the 15th sample was correct, the statement “You correctly predicted squamous cell on the previous patient” was appended to the end of the Basic prompt for the 16th sample. 

Explanation: The Explanation prompt is an aggregation of the Basic prompt with an injected explanation of why GPT made the previous prediction of SCC or AD. In addition to the prediction, GPT outputs an explanation of why it made that prediction which is then parsed and injected into the next sample’s prompt. Like with Feedback, this repeats for every prediction such that the next explanation replaces the previous one. For example, if GPT predicts SCC for the 15th sample, it may give an explanation such as, “The patient has high expression of genes such as *Gene1*, *Gene2*, *Gene3*, and *Gene4* which are keratin genes typically overexpressed in squamous cell carcinoma. Additionally, the high expression of *Gene5* and *Gene6*, which are known to be associated with squamous cell lung cancer, further supports this diagnosis.” This explanation is then appended to the end of the Basic prompt for the 16th sample.

Feedback and Explanation: The Feedback and Explanation prompt is an aggregation of the Basic, Feedback, and Explanation prompts. For a prediction, the feedback statement and explanation are both appended to the end of the Basic prompt. 

Fixed (temperature = 0): The Fixed (temperature = 0) prompt is an aggregation of the Basic prompt with a passage generated by GPT outlining the most important genes for SCC and AD prediction. First, the training samples are fed into the GPT Explanation model which generates a dataset containing 40 predictions and the associated 40 explanations. The explanations are then concatenated and fed into a GPT model using temperature = 0 with a prompt instructing it to summarize the 40 explanations. This summary, containing an explanation of why it makes predictions of SCC and AD, is then appended to the end of the Basic prompt. It remains the same across all testing samples, hence the name “Fixed”. 

Fixed (temperature = 0.5): The Fixed (temperature = 0.5) prompt is the same as the Fixed (temperature = 0) prompt with the only difference being the GPT summarizing model having a temperature of 0.5 instead of 0. 

Fixed (temperature = 1): The Fixed (temperature = 1) prompt is the same as the Fixed (temperature = 0) and Fixed (temperature = 0.5) prompts with the only difference being the GPT summarizing model having a temperature of 1. 

### 2.2. Kidney Cancer Prompts (Supplementary Materials)

*Basic*: The Basic prompt is the exact same as the lung cancer Basic prompt, with the only difference being the goal of diagnosing kidney cancer patients with ChRCC, CCRCC, or PRCC rather than lung cancers.

Fixed (temperature = 0): The Fixed (temperature = 0) prompt is the exact same as the lung cancer Fixed (temperature = 0) prompt except applied to the kidney cancer prediction task and training data. 

Fixed (temperature = 1): The Fixed (temperature = 1) prompt is the exact same as the lung cancer Fixed (temperature = 1) prompt except applied to the kidney cancer prediction task and training data. 

Experimental: The Experimental prompt is an aggregation of the kidney cancer Basic prompt with a list of genes that were statistically determined to be helpful for the prediction of ChRCC, CCRCC, or PRCC. Using Tukey’s test, the gene expression counts for each kidney cancer label are compared for each gene. Tukey’s returns the statistical significance for each of the three comparisons (ChRCC–CCRCC, ChRCC–PRCC, and CCRCC–PRCC), and each gene that has at least 2 significant comparisons is added to a list. Of the 431 genes in the kidney cancer dataset, 272 met these criteria. These 272 genes are then grouped based on each gene’s expression level of high or low (above the median or below the median, respectively) within each kidney cancer class and each is processed into a string. For example, the string, “Gene1: low ChRCC, high CCRCC, high PRCC”, means Gene1 has low expression in patients with ChRCC, high expression in patients with CCRCC, and high expression in patients with PRCC. The 272 processed strings are then appended to the end of the Basic prompt with a description of what these strings mean. 

#### Modeling

A HIPAA-secure OpenAI gpt-4 8k (Version 0613) API endpoint was used for all LLM experiments [15]. At the time of this experiment, there were no other accessible models that had a large enough context window to support the large context inputs, so all LLM experiments were limited to gpt-4. The traditional machine learning models selected were Logistic Regression, Support Vector Machines (SVM), Random Forest, LightGBM, and XGBoost. These were chosen due to their variability in how their underlying algorithms make predictions. All models used default parameters—there was no hyperparameter tuning performed. The metrics used for evaluation were precision, recall, and F1-score.

As aforementioned, the train/test split sample counts for lung cancer and kidney cancer datasets are 40:1000 and 60:969, respectively, which equates to a training dataset size of 3.8% and 5.8% of each cancer type’s dataset. Such a small ratio between train and test datasets is untraditional. However, this was a central part of the experimental design of this study. There are technological differences between traditional machine learning models and LLMs. The traditional models chosen were not pre-trained on a corpus of data; they required several training samples that mirror the type of data and structure of the testing samples. This is contrary to LLMs which are pre-trained on a massive corpus of text-based data and do not allow for further training on domain-specific data in the way traditional models are trained. The global performance of an LLM may be very good, but for specific task-oriented problems it may fall short. While fine tuning LLMs attempts to resolve this, it can be very expensive monetarily (and for other resources) and have mixed results. The performance of LLMs on specific tasks is thus dependent on the information in user prompts which are subject to context window limitations. Testing data must be fed to the LLM one query at a time, and to ensure the LLM understands the specific task being asked of it, the instructions and guidelines must be included in each query. When information in the user prompt is pushed out of the context window by subsequent queries, the LLM can no longer reliably remember it and it is lost. 

These fundamental differences of the traditional and LLM models make it necessary to level the playing field. Reducing the amount of training data fed into the traditional models and using default parameters will negatively impact model performance, but given the LLMs’ lack of further domain-specific training and one-query-at-a-time testing structure, this keeps it as close to an apples-to-apples comparison as possible. 

## 3. Results

### 3.1. Lung Cancer

**Figure 2:**
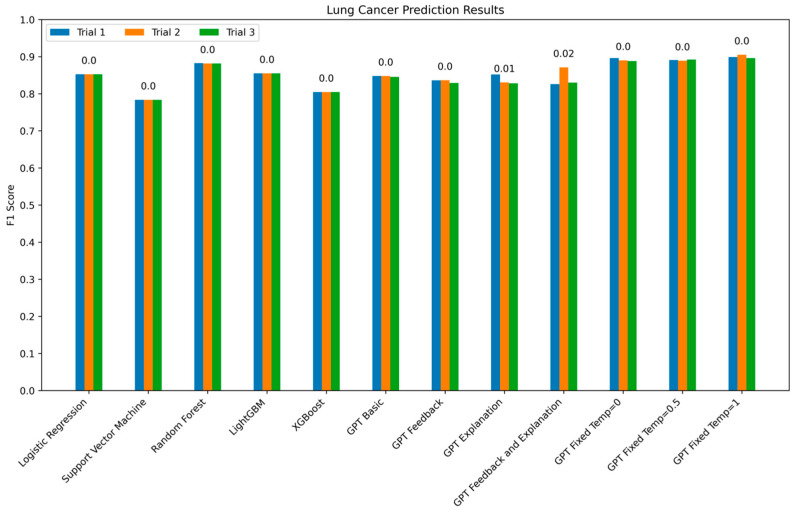
Lung cancer prediction results by model by trial. Each model was run three times to assess the consistency of prediction results. GPT Explanation and GPT Feedback and Explanation are the only two models that had trials with noticeably different results regarding performance. The overall best performing model was GPT Fixed temperature = 1. The standard deviations are shown above the bars for each model.

Each model was asked to predict 1000 non-small cell lung cancer samples as either SCC or AD. This experiment was repeated three times for each cancer (referred to as Trials 1, 2, and 3 in Figure 2) to determine variability from run to run. The best overall model was our GPT Fixed at Temp = 1 which outperformed all of the standard machine learning methods given the same amount of training data. This model had a fixed summary given with each prediction task which included genes and their expression levels that GPT determined were highly predictive of SCC and ADD. Importantly, providing input feedback did not improve performance. This demonstrates that simply providing additional information and training to the model does not inherently improve the model. What matters is the type of training data rather than the amount of training data for predictive tasks in genetic research using AI.

### 3.2. Kidney Cancer

**Figure 3:**
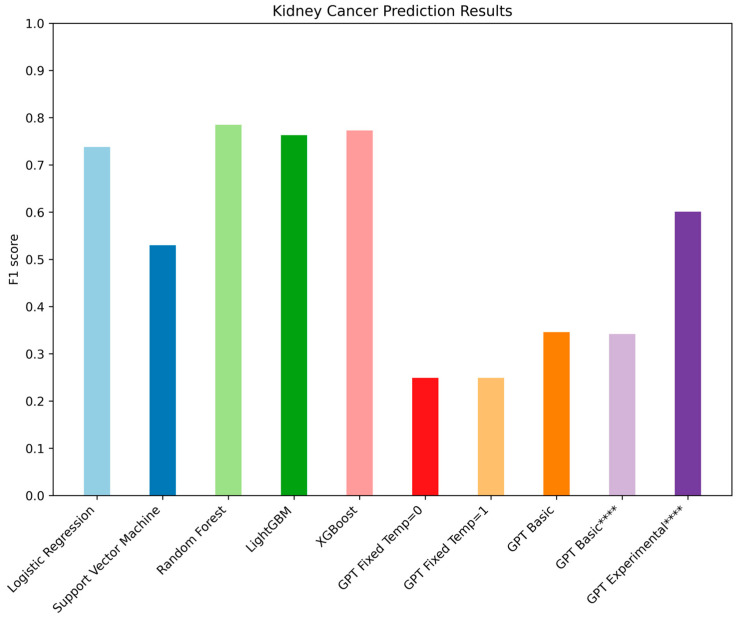
F1 scores across all ML and generative models in kidney cancer tests. **** These models were run at a different time then the rest of the experiments. The similarity between both sets of GPT Basic models shows that the difference between the run times is minimal. Overall, Random Forest was the best model, with GPT Experimental being the best of the GPT models.

In contrast to the lung cancer dataset and experiment, the kidney cancer dataset consists of three subtypes rather than two. The kidney cancer sample groups are ChRCC, CCRCC, or PRCC. The results of each model can be found in Figure 3 and Table 1.

## 4. Discussion

The overall goal of our project was to evaluate the efficacy of GPT-based models at analyzing real world gene expression data and using that information to make an evidence-based prediction. We performed two experiments, one on lung cancer data and the other on kidney cancer data.

### 4.1. Lung Cancer Experiments

The lung cancer experiments were overall easier predictions to make compared to the kidney cancer dataset. First, there are only two subtypes within the lung cancer dataset rather than three. Second, the biological differences between lung squamous cell carcinoma and lung adenocarcinoma are more distinct given that they are fundamentally different base cell types, making their gene expression profiles potentially easier to separate. Our results indicate that GPT with a fixed prompt at temperature 1 was the best performing model as measured by overall F1 score. This is surprising given that LLMs have not been designed to perform this kind of predictive analytic function. These LLMs were designed and built for the purpose of using human-written speech or text language as input rather than gene expression profiling data and were designed to generate text-based output rather than perform predictions. Regardless of the design intention, it appears as though for some gene expression questions GPT may be an interesting avenue for further research.

One important aspect of the multiple trial setup we were able to perform in our lung cancer experiments was to observe how consistent GPT-based model predictions were across trial runs. Surprisingly, GPT models performed remarkably consistently even when at a higher temperature. This is surprising as the higher temperature should make the models have a higher variability in what they select as the optimal output. However, we observed the most variability in trial-to-trial output from GPT models that were leveraging the explanation-style prompt. The only two GPT models with high variability both used this style of prompt, leading us to believe that this style of prompt may both increase run-to-run variability and decrease overall model performance as compared to a fixed prompt. 

### 4.2. Kidney Cancer Experiments

In these experiments, we were more interested in diving into how the GPT-based models’ predictions were performing rather than the consistency of the predictions across trials. Our results indicate two major takeaways; first, traditional machine learning algorithms significantly outperformed GPT-based models in predicting kidney cancer subtypes, and second, the experimental prompt type was the best performing GPT model.

Of all of the traditional machine learning algorithms tested, only the Support Vector Machine performed worse than the best GPT model as measured by overall F1 score. The GPT fixed models predicted every case as belonging to the same subgroup CCRCC, and both GPT basic models predicted no patients for the PRCC subgroup. Only the GPT experimental model appears to do a somewhat reasonable job at making predictions based on the gene expression profiles rather than purely based off of the differences in population frequency between the three subtypes. This demonstrates that GPT models are incredibly prompt sensitive when it comes to predictive analytics questions using gene expression data.

### 4.3. Cost

It is important to bring up one of the largest limiting factors in both our experimental design and of the broader use of large language models in performing gene expression analyses: cost. The overall cost of running the experiments provided in this manuscript greatly exceeds what would be expected of a normal natural language processing experiment. This cost is so high due to the large number of tokens that are needed to represent a small fraction of the human transcriptome. This makes the cost of running the GPT models exorbitant, especially when there are effectively free alternatives such as XGBoost and Random Forest. These more traditional ML models perform as well or better than the GPT models on the lung and kidney cancer datasets while being effectively free to run. This is in stark contrast to the GPT-based models, whose cost makes repeating trials cost prohibitive.

## 5. Conclusions

Our research indicates that there is great potential in leveraging transformer-based LLM models for analyzing and interpreting gene expression profiling data, but that there is much work that would still need to be done to bring these models up to current machine learning model standards. The ability of LLM models to interpret what gene expression data mean and use that data to make accurate predictions on cancer subtypes appears to be largely based on the framing style of the prompt. This indicates that future research should focus on optimizing gene expression analysis prompts rather than training the models to better understand what the genes themselves are or represent. It is important to understand and continuously evaluate how new LLM models and technologies perform on various tasks to understand how they can be leveraged. Given these models’ ability to extract gene expression information from text, it may be possible to create all-in-one medical AI pipelines that are able to extract, analyze, and interpret genetic information to help advance predictive and personalized medicine.

## Figures and Tables

**Table 1:** Precision, recall, and F1 Scores for all kidney cancer models.

Model	ChRCC Precision	CCRCC Precision	PRCC Precision	ChRCC Recall	CCRCC Recall	PRCC Recall	ChRCC F1	CCRCC F1	PRCC F1	Overall F1
Logistic Regression	0.407	0.887	0.911	0.912	0.832	0.700	0.563	0.859	0.792	0.738
Support Vector Machine	0.260	0.719	0.871	0.582	0.829	0.313	0.359	0.770	0.460	0.530
Random Forest	0.422	0.961	0.936	0.978	0.837	0.817	0.589	0.895	0.873	0.786
LightGBM	0.397	0.948	0.926	0.978	0.824	0.774	0.565	0.882	0.843	0.763
XGBoost	0.413	0.948	0.925	0.967	0.827	0.799	0.579	0.883	0.857	0.773
GPT Basic	0.325	0.610	0.000	0.275	0.945	0.000	0.298	0.741	0.000	0.346
GPT Fixed Temp = 0	0.000	0.597	0.000	0.000	1.000	0.000	0.000	0.748	0.000	0.249
GPT Fixed Temp = 1	0.000	0.597	0.000	0.000	1.000	0.000	0.000	0.748	0.000	0.249
GPT Basic ****	0.333	0.604	0.000	0.259	0.941	0.000	0.292	0.736	0.000	0.342
GPT Experimental ****	0.283	0.827	0.909	0.963	0.773	0.412	0.437	0.799	0.567	0.601

**** These models were run at a different time then the rest of the experiments. The similarity between both sets of GPT Basic models shows that the difference between the run times is minimal. Overall, Random Forest was the best model, with GPT Experimental being the best of the GPT models.

## Data Availability

All data used in this study are publicly available at The Cancer Genome Atlas (TCGA). The adenocarcinoma cohort can be found at https://portal.gdc.cancer.gov/projects/TCGA-LUAD accessed on 24 August 2023. The squamous cell carcinoma cohort can be found at https://portal.gdc.cancer.gov/projects/TCGA-LUSC accessed on 24 August 2023. The kidney chromophobe cohort can be found at https://portal.gdc.cancer.gov/projects/TCGA-KICH accessed on 24 August 2023. The kidney renal cell carcinoma cohort can be found at https://portal.gdc.cancer.gov/projects/TCGA-KIRC accessed on 24 August 2023. Kidney renal papillary cell carcinoma cohort can be found at https://portal.gdc.cancer.gov/projects/TCGA-KIRP accessed on 24 August 2023. To extract a dataset, save the cohort, navigate to the Repository tab, select the saved cohort, and under the Experimental Strategy filter select “RNA-Seq”, under the Data Type filter select “Gene Expression Quantification”, and under the Data Format select “tsv”; finally, add all files to the cart for download.

## Supplementary Materials

The following supporting information can be downloaded at: https://www.mdpi.com/article/10.3390/jpm14101022/s1, Supplementary Materials A: Lung cancer GPT prompts; Supplementary Materials B: Kidney cancer GPT prompts.

## References

[1] Katsanis S.H., Katsanis N. (2013). Molecular genetic testing and the future of clinical genomics. Nat. Rev. Genet..

[2] Sperber N.R., Carpenter J.S., Cavallari L.H., Damschroder L.J., Cooper-DeHoff R.M., Denny J.C., Ginsburg G.S., Guan Y., Horowitz C.R., Levy K.D. (2017). Challenges and strategies for implementing genomic services in diverse settings: Experiences from the Implementing GeNomics In pracTicE (IGNITE) network. BMC Med. Genom..

[3] Patel V.L., Shortliffe E.H., Stefanelli M., Szolovits P., Berthold M.R., Bellazzi R., Abu-Hanna A. (2009). The coming of age of artificial intelligence in medicine. Artif. Intell. Med..

[4] Bommasani R., Hudson D.A., Adeli E., Altman R., Arora S., Von Arx S., Bernstein M.S., Bohg J., Bosselut A., Brunskill E. (2021). On the Opportunities and Risks of Foundation Models. arXiv.

[5] Azam M., Chen Y., Arowolo M.O., Liu H., Popescu M., Xu D. (2024). A Comprehensive Evaluation of Large Language Models in Mining Gene Interactions and Pathway Knowledge. bioRxiv.

[6] Pratt D., Hu M., Alkhairy S., Pillich R., Bachelder R., Ideker T. (2023). Evaluation of large language models for discovery of gene set function. arXiv.

[7] Gill J.K., Chetty M., Lim S., Hallinan J. (2024). Large language model based framework for automated extraction of genetic interactions from unstructured data. PLoS ONE.

[8] Toufiq M., Rinchai D., Bettacchioli E., Kabeer B.S.A., Khan T., Subba B., White O., Yurieva M., George J., Jourde-Chiche N. (2023). Harnessing large language models (LLMs) for candidate gene prioritization and selection. J. Transl. Med..

[9] Quazi S. (2022). Artificial intelligence and machine learning in precision and genomic medicine. Med. Oncol..

[10] Thieme A., Nori A., Ghassemi M., Bommasani R., Andersen T.O., Luger E. Foundation Models in Healthcare: Opportunities, Risks & Strategies Forward. Proceedings of the Extended Abstracts of the 2023 CHI Conference on Human Factors in Computing Systems.

[11] Consens M.E., Dufault C., Wainberg M., Forster D., Karimzadeh M., Goodarzi H., Theis F.J., Moses A., Wang B. (2023). To Transformers and Beyond: Large Language Models for the Genome. arXiv.

[12] Sherafatian M., Arjmand F. (2019). Decision tree-based classifiers for lung cancer diagnosis and subtyping using TCGA miRNA expression data. Oncol Lett..

[13] Ali A.M., Zhuang H., Ibrahim A., Rehman O., Huang M., Wu A. (2018). A machine learning approach for the classification of kidney cancer subtypes using miRNA genome data. Appl. Sci..

[14] Liljedahl H., Karlsson A., Oskarsdottir G.N., Salomonsson A., Brunnström H., Erlingsdottir G., Jönsson M., Isaksson S., Arbajian E., Ortiz-Villalón C. (2021). A gene expression-based single sample predictor of lung adenocarcinoma molecular subtype and prognosis. Int. J. Cancer.

[15] Achiam J., Adler S., Agarwal S., Ahmad L., Akkaya I., Aleman F.L., Almeida D., Altenschmidt J., Altman S., OpenAI (2023). GPT-4 Technical Report. arXiv.

