# Supplementary material for: Evaluating Generative AI’s Ability to Identify Cancer Subtypes in Publicly Available Structured Genetic Datasets
Source: J Pers Med. 2024 Sep 25;14(10):1022. doi: 10.3390/jpm14101022 (PMC11508243; doi:10.3390/jpm14101022)
Supplement: Supplementary file 1 [file jpm-14-01022-s001.zip › jpm-3168922-supplementary.pdf]

Supplementary Materials A: *Lung Cancer Prompts*

System Prompt:
"You are a smart and intelligent geneticist agent, your goal is to diagnose a lung cancer patient as having squamous cell or adenocarcinoma lung cancer based on their gene expression. I will provide you a lung cancer patient's gene expression in the form of a list of gene-count pairs and you will tell me if the patient has squamous cell or adenocarcinoma lung cancer."
user_prompt = "Are you clear about your role?"
assistant_prompt = "Sure, I'm ready to help you with your genetic expression task. Please provide me with the necessary information to get started."

Basic Prompt

"Gene expression is the process by which the information encoded in a gene is used to direct the assembly of a protein molecule. It's essentially how a gene gets turned into a functional product. Gene expression can be measured in a number of ways, with one common method being 'counting' the number of mRNA molecules a particular gene produces - i.e., gene expression counts.\n"

"\n"

"If a gene has a high expression count, it means that it's being actively transcribed and translated more than other genes. This usually means that the protein it codes for is in high demand within the cell or tissue. For example, genes responsible for cell growth may be highly expressed in a rapidly growing cell, while genes associated with a specific function may be highly expressed in cells that serve that function. Low gene expression means the opposite; the gene is not frequently transcribed into mRNA. It might mean that the protein product (if the gene codes for one) is not needed in large amounts within the specific cell type, tissue, or under the specific condition in which the observation is being made.\n"

"\n"

"If a gene that's typically not highly expressed suddenly shows high expression, or a gene that's typically highly expressed suddenly shows low expression, it can be a sign of certain diseases. For instance, many cancer cells have alterations in gene expression, where genes that stimulate cell division are overexpressed, leading to uncontrolled cell growth.\n"

"\n"

"Output format:\n"

"{{'diagnosis': 'squamous cell' or 'adenocarcinoma'}}\n"

"Below are gene-count pairs that represent the gene expression for a single patient with lung cancer. Please diagnoses them with squamous cell or adenocarcinoma.\n"

"Text: {}\n"

"Output: "

Feedback Prompt

"Gene expression is the process by which the information encoded in a gene is used to direct the assembly of a protein molecule. It's essentially how a gene gets turned into a functional product. Gene expression can be measured in a number of ways, with one common method being 'counting' the number of mRNA molecules a particular gene produces - i.e., gene expression counts.\n"

"\n"

"If a gene has a high expression count, it means that it's being actively transcribed and translated more than other genes. This usually means that the protein it codes for is in high demand within the cell or tissue. For example, genes responsible for cell growth may be highly expressed in a rapidly growing cell, while genes associated with a specific function may be highly expressed in cells that serve that function. Low gene

expression means the opposite; the gene is not frequently transcribed into mRNA. It might mean that the protein product (if the gene codes for one) is not needed in large amounts within the specific cell type, tissue, or under the specific condition in which the observation is being made.\n"

"\n"

"If a gene that's typically not highly expressed suddenly shows high expression, or a gene that's typically highly expressed suddenly shows low expression, it can be a sign of certain diseases. For instance, many cancer cells have alterations in gene expression, where genes that stimulate cell division are overexpressed, leading to uncontrolled cell growth.\n"

"\n"

"Here is some context to help you better make the diagnosis of squamous cell or adenocarcinoma:\n"

"{0}\n"

"\n"

"Output format:\n"

"{{'diagnosis': 'squamous cell' or 'adenocarcinoma'}}\n"

"\n"

"Below are gene-count pairs that represent the gene expression for a single patient with lung cancer. Please diagnoses them with squamous cell or adenocarcinoma.\n"

"Text: {1}\n"

"Output: "

Explanation Prompt

"Gene expression is the process by which the information encoded in a gene is used to direct the assembly of a protein molecule. It's essentially how a gene gets turned into a functional product. Gene expression can be measured in a number of ways, with one common method being 'counting' the number of mRNA molecules a particular gene produces - i.e., gene expression counts.\n"

"\n"

"If a gene has a high expression count, it means that it's being actively transcribed and translated more than other genes. This usually means that the protein it codes for is in high demand within the cell or tissue. For example, genes responsible for cell growth may be highly expressed in a rapidly growing cell, while genes associated with a specific function may be highly expressed in cells that serve that function. Low gene expression means the opposite; the gene is not frequently transcribed into mRNA. It might mean that the protein product (if the gene codes for one) is not needed in large amounts within the specific cell type, tissue, or under the specific condition in which the observation is being made.\n"

"\n"

"If a gene that's typically not highly expressed suddenly shows high expression, or a gene that's typically highly expressed suddenly shows low expression, it can be a sign of certain diseases. For instance, many cancer cells have alterations in gene expression, where genes that stimulate cell division are overexpressed, leading to uncontrolled cell growth.\n"

"\n"

"Here is some context to help you better make the diagnosis of squamous cell or adenocarcinoma:\n"

"{0}\n"

"\n"

"Output format:\n"

"{{'diagnosis': 'squamous cell' or 'adenocarcinoma'}}\n"

"\n"

"Below are gene-count pairs that represent the gene expression for a single patient with lung cancer. Please diagnoses them with squamous cell or adenocarcinoma.\n"

"Text: {1}\n"

"Output: "

Feedback and Explanation

"Gene expression is the process by which the information encoded in a gene is used to direct the assembly of a protein molecule. It's essentially how a gene gets turned into a functional product. Gene expression can be measured in a number of ways, with one common method being 'counting' the number of mRNA molecules a particular gene produces - i.e., gene expression counts.\n"

"\n"

"If a gene has a high expression count, it means that it's being actively transcribed and translated more than other genes. This usually means that the protein it codes for is in high demand within the cell or tissue. For example, genes responsible for cell growth may be highly expressed in a rapidly growing cell, while genes associated with a specific function may be highly expressed in cells that serve that function. Low gene expression means the opposite; the gene is not frequently transcribed into mRNA. It might mean that the protein product (if the gene codes for one) is not needed in large amounts within the specific cell type, tissue, or under the specific condition in which the observation is being made.\n"

"\n"

"If a gene that's typically not highly expressed suddenly shows high expression, or a gene that's typically highly expressed suddenly shows low expression, it can be a sign of certain diseases. For instance, many cancer cells have alterations in gene expression, where genes that stimulate cell division are overexpressed, leading to uncontrolled cell growth.\n"

"\n"

"Here is some context to help you better make the diagnosis of squamous cell or adenocarcinoma:\n"

"{0}\n"

"\n"

"{1}"

"\n"

"Output format:\n"

"{{'diagnosis': 'squamous cell' or 'adenocarcinoma', 'explanation': explanation for diagnosis}}\n"

"\n"

"Below are gene-count pairs that represent the gene expression for a single patient with lung cancer. Please diagnoses them with squamous cell or adenocarcinoma, give the explanation for why you made that diagnosis, and output it in the given output format.\n"

"Text: {2}\n"

"Output: "

Fixed Temp=0

"Gene expression is the process by which the information encoded in a gene is used to direct the assembly of a protein molecule. It's essentially how a gene gets turned into a functional product. Gene expression can be measured in a number of ways, with one common method being 'counting' the number of mRNA molecules a particular gene produces - i.e., gene expression counts.\n"

"\n"

"If a gene has a high expression count, it means that it's being actively transcribed and translated more than other genes. This usually means that the protein it codes for is in high demand within the cell or tissue. For example, genes responsible for cell growth may be highly expressed in a rapidly growing cell, while genes associated with a specific function may be highly expressed in cells that serve that function. Low gene expression means the opposite; the gene is not frequently transcribed into mRNA. It might mean that the protein product (if the gene codes for one) is not needed in large amounts within the specific cell type, tissue, or under the specific condition in which the observation is being made.\n"

"\n"

"If a gene that's typically not highly expressed suddenly shows high expression, or a gene that's typically highly expressed suddenly shows low expression, it can be a sign of certain diseases. For instance, many cancer cells have alterations in gene expression, where genes that stimulate cell division are overexpressed, leading to uncontrolled cell growth.\n"

"\n"

"Here is some context to help you better make the diagnosis of squamous cell or adenocarcinoma:\n"

"The diagnosis of squamous cell lung cancer or adenocarcinoma is primarily based on the expression levels of specific genes. \n\nFor squamous cell lung cancer, high expression of genes such as KRT5, KRT6A, KRT6B, KRT14, KRT16, KRT19, CEACAM6, LGALS3, and MMP1 is observed. These genes are typically overexpressed in squamous lung cancers. KRT5 and KRT6A are cytokeratins usually found in cells of squamous epithelium, their high expression suggests a squamous cell phenotype. The overexpression of CEACAM6, which is associated with cell adhesion a crucial factor in cancer metastasis and MMP1, linked with extracellular matrix degradation aiding in cancer cell invasion and metastasis, further supports the squamous cell diagnosis.\n\nOn the other hand, adenocarcinoma is diagnosed when there is high expression of genes like CEACAM5, CEACAM6, REG4, and AGR2, which are usually upregulated in adenocarcinomas. The high expression of CEACAM6, a gene that is often overexpressed in adenocarcinoma, and relatively low expression of KRT5 and KRT6A, which are typically associated with squamous cell carcinoma, are indicative of adenocarcinoma lung cancer. \n\nIn summary, the diagnosis of squamous cell lung cancer or adenocarcinoma is determined by the relative expression levels of a set of genes, with KRT5, KRT6A, and CEACAM6 being key markers. High expression of KRT5 and KRT6A suggests squamous cell lung cancer, while high expression of CEACAM6 suggests adenocarcinoma.\n"

"\n"

"Output format:\n"

"{{'diagnosis': 'squamous cell' or 'adenocarcinoma'}}\n"

"Below are gene-count pairs that represent the gene expression for a single patient with lung cancer. Please diagnoses them with squamous cell or adenocarcinoma.\n"

"Text: {1}\n"

"Output: "

Fixed Temp=0.5

"Gene expression is the process by which the information encoded in a gene is used to direct the assembly of a protein molecule. It's essentially how a gene gets turned into a functional product. Gene expression can be measured in a number of ways, with one common method being 'counting' the number of mRNA molecules a particular gene produces - i.e., gene expression counts.\n"

"\n"

"If a gene has a high expression count, it means that it's being actively transcribed and translated more than other genes. This usually means that the protein it codes for is in high demand within the cell or tissue. For example, genes responsible for cell growth may be highly expressed in a rapidly growing cell, while genes associated with a specific function may be highly expressed in cells that serve that function. Low gene expression means the opposite; the gene is not frequently transcribed into mRNA. It might mean that the protein product (if the gene codes for one) is not needed in large amounts within the specific cell type, tissue, or under the specific condition in which the observation is being made.\n"

"\n"

"If a gene that's typically not highly expressed suddenly shows high expression, or a gene that's typically highly expressed suddenly shows low expression, it can be a sign of certain diseases. For instance, many cancer cells have alterations in gene expression, where genes that stimulate cell division are overexpressed, leading to uncontrolled cell growth.\n"

"\n"

"Here is some context to help you better make the diagnosis of squamous cell or adenocarcinoma:\n"

"The diagnosis of squamous cell lung cancer or adenocarcinoma lung cancer in patients is primarily based on the expression levels of certain genes. \n\nFor squamous cell lung cancer, high expression of genes such as KRT5, KRT6A, KRT6B, KRT14, KRT16, KRT19, CEACAM6,

LGALS3, and MMP1 is observed. These genes are typically overexpressed in squamous lung cancers. KRT5, KRT6A, and KRT6B encode for cytokeratins usually found in cells of squamous epithelium. Overexpression of CEACAM6 is associated with cell adhesion, a crucial factor in cancer metastasis. MMP1 is associated with extracellular matrix degradation aiding in cancer cell invasion and metastasis. High level expressions of these genes generally indicate squamous cell lung cancer.\n\nOn the other hand, for adenocarcinoma lung cancer, high expression of genes such as CEACAM5, CEACAM6, and REG4 is seen. These genes are typically upregulated in adenocarcinomas. CEACAM6 is a gene that is often overexpressed in this type of cancer. Other supportive evidence includes the overexpression of genes like CD63, SERPINA1, and PKM which are typically associated with adenocarcinoma. \n\nIn cases where the expression of squamous cell carcinoma genes is low and adenocarcinoma genes is high, the patient is more likely to have adenocarcinoma lung cancer and vice versa. This differential gene expression profiling aids in distinguishing between squamous cell lung cancer and adenocarcinoma lung cancer.\n"

"\n"

"Output format:\n"

"{{'diagnosis': 'squamous cell' or 'adenocarcinoma'}}\n"

"Below are gene-count pairs that represent the gene expression for a single patient with lung cancer. Please diagnoses them with squamous cell or adenocarcinoma.\n"

"Text: {1}\n"

"Output: "

Fixed Temp=1

"Gene expression is the process by which the information encoded in a gene is used to direct the assembly of a protein molecule. It's essentially how a gene gets turned into a functional product. Gene expression can be measured in a number of ways, with one common method being 'counting' the number of mRNA molecules a particular gene produces - i.e., gene expression counts.\n"

"\n"

"If a gene has a high expression count, it means that it's being actively transcribed and translated more than other genes. This usually means that the protein it codes for is in high demand within the cell or tissue. For example, genes responsible for cell growth may be highly expressed in a rapidly growing cell, while genes associated with a specific function may be highly expressed in cells that serve that function. Low gene expression means the opposite; the gene is not frequently transcribed into mRNA. It might mean that the protein product (if the gene codes for one) is not needed in large amounts within the specific cell type, tissue, or under the specific condition in which the observation is being made.\n"

"\n"

"If a gene that's typically not highly expressed suddenly shows high expression, or a gene that's typically highly expressed suddenly shows low expression, it can be a sign of certain diseases. For instance, many cancer cells have alterations in gene expression, where genes that stimulate cell division are overexpressed, leading to uncontrolled cell growth.\n"

"\n"

"Here is some context to help you better make the diagnosis of squamous cell or adenocarcinoma:\n"

"Summarizing, the diagnosis of squamous cell lung cancer or adenocarcinoma can often be made by identifying the overexpression of key genes associated with each type: \n\n- For squamous cell lung cancer, keratin (KRT) genes like KRT5, KRT6A, KRT6B, KRT14, KRT16, KRT19, LGALS3, and MMP1 are typically overly expressed. Cytokeratins, found in squamous epithelium cells, support a squamous cell lung cancer diagnosis when highly expressed. \n\n- On the other hand, elevated levels of CEACAM5, CEACAM6, and AGR2 genes, as well as REG4 and SERPINA1, are common characteristics of adenocarcinoma lung cancer. \n\nThe patients revealed high expression of KRT genes for squamous cell carcinoma patients and, conversely, high expression of CEACAM genes for those with adenocarcinoma. It is critical to note that low expressions of certain gene markers from one type do not automatically confirm the other type. It is a comparative evaluation of both the high expressions of certain gene markers and the lower expressions of other relevant gene markers that leads to an accurate diagnosis. \n\nIn brief, squamous cell lung cancer diagnosis is based on high expression of KRT genes and low expression adenocarcinoma markers, while adenocarcinoma is diagnosed on high expression of CEACAM genes and low expression of squamous cell carcinoma markers.\n"

"\n"

"Output format:\n"

"{{'diagnosis': 'squamous cell' or 'adenocarcinoma'}}\n"

"Below are gene-count pairs that represent the gene expression for a single patient with lung cancer. Please diagnoses them with squamous cell or adenocarcinoma.\n"

"Text: {1}\n"

"Output: "

Supplementary Materials *B: Kidney Cancer Prompts*

System Prompt:

"You are a smart and intelligent geneticist agent, your goal is to diagnose a kidney cancer patient as having chromophobe renal cell carcinoma or clear cell renal cell carcinoma or papillary renal cell carcinoma based on their gene expression. Chromophobe renal cell carcinoma will hereafter be known as CHRCC. Clear cell renal cell carcinoma will hereafter be known as CCRCC. Papillary renal cell carcinoma will hereafter be known as PRCC. I will provide you a kidney cancer patient's gene expression in the form of a list of gene-count pairs and you will tell me if the patient has CHRCC or CCRCC or PRCC using your expertise in gene expression."

Basic

"Gene expression is the process by which the information encoded in a gene is used to direct the assembly of a protein molecule. It's essentially how a gene gets turned into a functional product. Gene expression can be measured in a number of ways, with one common method being 'counting' the number of mRNA molecules a particular gene produces - i.e., gene expression counts.\n"

"\n"

"If a gene has a high expression count, it means that it's being actively transcribed and translated more than other genes. This usually means that the protein it codes for is in high demand within the cell or tissue. For example, genes responsible for cell growth may be highly expressed in a rapidly growing cell, while genes associated with a specific function may be highly expressed in cells that serve that function. Low gene expression means the opposite; the gene is not frequently transcribed into mRNA. It might mean that the protein product (if the gene codes for one) is not needed in large amounts within the specific cell type, tissue, or under the specific condition in which the observation is being made.\n"

"\n"

"If a gene that's typically not highly expressed suddenly shows high expression, or a gene that's typically highly expressed suddenly shows low expression, it can be a sign of certain diseases. For instance, many cancer cells have alterations in gene expression, where genes that stimulate cell division are overexpressed, leading to uncontrolled cell growth.\n"

"\n"

"Output format:\n"

"{{'diagnosis': 'chromophobe renal cell carcinoma' or 'clear cell renal cell carcinoma' or 'papillary renal cell carcinoma'}}\n"

"Below are gene-count pairs that represent the gene expression for a single patient with lung cancer. Please diagnoses them with chromophobe renal cell carcinoma or clear cell renal cell carcinoma or papillary renal cell carcinoma.\n"

"Text: {}\n"

"Output: "

Feedback

"Gene expression is the process by which the information encoded in a gene is used to direct the assembly of a protein molecule. It's essentially how a gene gets turned into a functional product. Gene expression can be measured in a number of ways, with one common method being 'counting' the number of mRNA molecules a particular gene produces - i.e., gene expression counts.\n"

"\n"

"If a gene has a high expression count, it means that it's being actively transcribed and translated more than other genes. This usually means that the protein it codes for is in high demand within the cell or tissue. For example, genes responsible for cell growth may be highly expressed in a rapidly growing cell, while genes associated with a specific function may be highly expressed in cells that serve that function. Low gene expression means the opposite; the gene is not frequently transcribed into mRNA. It might mean that the protein product (if the gene codes for one) is not needed in large amounts within the specific cell type, tissue, or under the specific condition in which the observation is being made.\n"

"\n"

"If a gene that's typically not highly expressed suddenly shows high expression, or a gene that's typically highly expressed suddenly shows low expression, it can be a sign of certain diseases. For instance, many cancer cells have alterations in gene expression, where genes that stimulate cell division are overexpressed, leading to uncontrolled cell growth.\n"

"\n"

"Here is some context to help you better make the diagnosis of chromophobe renal cell carcinoma or clear cell renal cell carcinoma or papillary renal cell carcinoma:\n"

"{0}\n"

"\n"

"Output format:\n"

"{{'diagnosis': 'chromophobe renal cell carcinoma' or 'clear cell renal cell carcinoma' or 'papillary renal cell carcinoma'}}\n"

"\n"

"Below are gene-count pairs that represent the gene expression for a single patient with lung cancer. Please diagnoses them with chromophobe renal cell carcinoma or clear cell renal cell carcinoma or papillary renal cell carcinoma.\n"

"Text: {1}\n"

"Output: "

Explanation

"Gene expression is the process by which the information encoded in a gene is used to direct the assembly of a protein molecule. It's essentially how a gene gets turned into a functional product. Gene expression can be measured in a number of ways, with one common method being 'counting' the number of mRNA molecules a particular gene produces - i.e., gene expression counts.\n"

"\n"

"If a gene has a high expression count, it means that it's being actively transcribed and translated more than other genes. This usually means that the protein it codes for is in high demand within the cell or tissue. For example, genes responsible for cell growth may be highly expressed in a rapidly growing cell, while genes associated with a specific function may be highly expressed in cells that serve that function. Low gene expression means the opposite; the gene is not frequently transcribed into mRNA. It might mean that the protein product (if the gene codes for one) is not needed in large amounts within the specific cell type, tissue, or under the specific condition in which the observation is being made.\n"

"\n"

"If a gene that's typically not highly expressed suddenly shows high expression, or a gene that's typically highly expressed suddenly shows low expression, it can be a sign of certain diseases. For instance, many cancer cells have alterations in gene expression, where genes that stimulate cell division are overexpressed, leading to uncontrolled cell growth.\n"

"\n"

"Here is some context to help you better make the diagnosis of chromophobe renal cell carcinoma or clear cell renal cell carcinoma or papillary renal cell carcinoma:\n"

"{0}\n"

"\n"

"Output format:\n"

"{{'diagnosis': 'chromophobe renal cell carcinoma' or 'clear cell renal cell carcinoma' or 'papillary renal cell carcinoma'}}\n"

"\n"

"Below are gene-count pairs that represent the gene expression for a single patient with lung cancer. Please diagnoses them with chromophobe renal cell carcinoma or clear cell renal cell carcinoma or papillary renal cell carcinoma.\n"

"Text: {1}\n"

"Output: "

Feedback and Explanation

"Gene expression is the process by which the information encoded in a gene is used to direct the assembly of a protein molecule. It's essentially how a gene gets turned into a functional product. Gene expression can be measured in a number of ways, with one common method being 'counting' the number of mRNA molecules a particular gene produces - i.e., gene expression counts.\n"

"\n"

"If a gene has a high expression count, it means that it's being actively transcribed and translated more than other genes. This usually means that the protein it codes for is in high demand within the cell or tissue. For example, genes responsible for cell growth may be highly expressed in a rapidly growing cell, while genes associated with a specific function may be highly expressed in cells that serve that function. Low gene expression means the opposite; the gene is not frequently transcribed into mRNA. It might mean that the protein product (if the gene codes for one) is not needed in large amounts within the specific cell type, tissue, or under the specific condition in which the observation is being made.\n"

"\n"

"If a gene that's typically not highly expressed suddenly shows high expression, or a gene that's typically highly expressed suddenly shows low expression, it can be a sign of certain diseases. For instance, many cancer cells have alterations in gene expression, where genes that stimulate cell division are overexpressed, leading to uncontrolled cell growth.\n"

"\n"

"Here is some context to help you better make the diagnosis of squamous cell or adenocarcinoma:\n"

"{0}\n"

"\n"

"{1}"

"\n"

"Output format:\n"

"{{'diagnosis': 'chromophobe renal cell carcinoma' or 'clear cell renal cell carcinoma' or 'papillary renal cell carcinoma', 'explanation': explanation for diagnosis}}\n"

"\n"

"Below are gene-count pairs that represent the gene expression for a single patient with lung cancer. Please diagnoses them with squamous cell or adenocarcinoma, give the explanation for why you made that diagnosis, and output it in the given output format.\n"

"Text: {2}\n"

"Output: "

Fixed Temp=0

"Gene expression is the process by which the information encoded in a gene is used to direct the assembly of a protein molecule. It's essentially how a gene gets turned into a functional product. Gene expression can be measured in a number of ways, with one common method being 'counting' the number of mRNA molecules a particular gene produces - i.e., gene expression counts.\n"

"\n"

"If a gene has a high expression count, it means that it's being actively transcribed and translated more than other genes. This usually means that the protein it codes for is in high demand within the cell or tissue. For example, genes responsible for cell growth may be highly expressed in a rapidly growing cell, while genes associated with a specific function may be highly expressed in cells that serve that function. Low gene expression means the opposite; the gene is not frequently transcribed into mRNA. It might mean that the protein product (if the gene codes for one) is not needed in large amounts within the specific cell type, tissue, or under the specific condition in which the observation is being made.\n"

"\n"

"If a gene that's typically not highly expressed suddenly shows high expression, or a gene that's typically highly expressed suddenly shows low expression, it can be a sign of certain diseases. For instance, many cancer cells have alterations in gene expression, where genes that stimulate cell division are overexpressed, leading to uncontrolled cell growth.\n"

"\n"

"Here is some context to help you better make the diagnosis of chromophobe renal cell carcinoma or clear cell renal cell carcinoma or papillary renal cell carcinoma:\n"

"The diagnosis of chromophobe renal cell carcinoma, clear cell renal cell carcinoma, or papillary renal cell carcinoma is primarily determined by the expression levels of specific genes. \n\nClear cell renal cell carcinoma (ccRCC) is typically characterized by high expression levels of genes such as CA9, CA12, VEGFA, CD9, NDRG1, and VIM. In particular, CA12 and VEGFA are often overexpressed, while genes like ATP6V0A4 and PKM, associated with chromophobe and papillary renal cell carcinomas, are usually not downregulated. \n\nChromophobe renal cell carcinoma is often associated with high expression levels of genes such as CDH16, AQP1, AQP2, and ATP6V0A4. However, the expression levels of genes associated with clear cell renal cell carcinoma, such as VEGFA and CA12, are usually not as high in patients with chromophobe renal cell carcinoma.\n\nPapillary renal cell carcinoma is often associated with high expression levels of genes such as KRT7 and GATM. However, the expression levels of genes associated with clear cell renal cell carcinoma, such as VEGFA and CA12, are usually not as high in patients with papillary renal cell carcinoma.\n\nIn summary, the diagnosis of these types of renal cell carcinoma is based on the relative expression levels of specific genes. High expression of CA12 and VEGFA typically indicates clear cell renal cell carcinoma, high expression of CDH16 and AQP2 suggests chromophobe renal cell carcinoma, and high expression of KRT7 and GATM points to papillary renal cell carcinoma. However, these are not absolute indicators, and a comprehensive genetic profile is necessary for a definitive diagnosis.\n"

"\n"

"Output format:\n"

"{{'diagnosis': 'chromophobe renal cell carcinoma' or 'clear cell renal cell carcinoma' or 'papillary renal cell carcinoma'}}\n"

"Below are gene-count pairs that represent the gene expression for a single patient with lung cancer. Please diagnoses them with chromophobe renal cell carcinoma or clear cell renal cell carcinoma or papillary renal cell carcinoma.\n"

"Text: {1}\n"

"Output: "

Fixed Temp=1

"Gene expression is the process by which the information encoded in a gene is used to direct the assembly of a protein molecule. It's essentially how a gene gets turned into a functional product. Gene expression can be measured in a number of ways, with one common method being 'counting' the number of mRNA molecules a particular gene produces - i.e., gene expression counts.\n"

"\n"

"If a gene has a high expression count, it means that it's being actively transcribed and translated more than other genes. This usually means that the protein it codes for is in high demand within the cell or tissue. For example, genes responsible for cell growth may be highly expressed in a rapidly growing cell, while genes associated with a specific function may be highly expressed in cells that serve that function. Low gene expression means the opposite; the gene is not frequently transcribed into mRNA. It might mean that the protein product (if the gene codes for one) is not needed in large amounts within the specific cell type, tissue, or under the specific condition in which the observation is being made.\n"

"\n"

"If a gene that's typically not highly expressed suddenly shows high expression, or a gene that's typically highly expressed suddenly shows low expression, it can be a sign of certain diseases. For instance, many cancer cells have alterations in gene expression, where genes that stimulate cell division are overexpressed, leading to uncontrolled cell growth.\n"

"\n"

"Here is some context to help you better make the diagnosis of chromophobe renal cell carcinoma or clear cell renal cell carcinoma or papillary renal cell carcinoma:\n"

"In summary, a clear pattern can be seen in the diagnoses of these three types of kidney cancers based on gene expression profiles:\n\nFor clear cell renal cell carcinoma (ccRCC), high expression level of genes such as CA12, NDRG1, CD9, VEGFA and VIM are typically observed. The elevated transcription level of genes CA9 and VEGFA is a key marker for ccRCC diagnosis. Additionally, relatively normal expressions of other genes typically associated with chromophobe or papillary renal cell carcinoma, such as CDH16, AQP2, and GATM tends to support a diagnosis of ccRCC.\n\nDiagnosis of chromophobe renal cell carcinoma is suggested by overexpression of CDH16, AQP2, and ATP6V0A4 genes. The lack of overexpression of genes CA12 and VEGFA—which are often found in ccRCC—and somewhat normal or lower expressions for papillary renal cell carcinoma markers suggest chromophobe renal cell carcinoma as the most likely diagnosis.\n\nPapillary renal cell carcinoma has less defined gene markers compared to the other two types of kidney cancer, but overexpression of genes such as GATM and KRT7 may suggest diagnosis. Relative downregulation or moderate expressions of genes evident in ccRCC and chromophobe renal cell carcinoma also support the diagnosis of papillary renal cell carcinoma.\n\nPlease note, in some cases, the gene expression profile might show markers common to more than one type of carcinoma, leading to more complex diagnoses. Overall, the diagnosis is made by combining gene expressions and their interactions, with frequent emphasis on key diagnostic genes.
\n"

"\n"

"Output format:\n"

"{{'diagnosis': 'chromophobe renal cell carcinoma' or 'clear cell renal cell carcinoma' or 'papillary renal cell carcinoma'}}\n"

"Below are gene-count pairs that represent the gene expression for a single patient with lung cancer. Please diagnoses them with chromophobe renal cell carcinoma or clear cell renal cell carcinoma or papillary renal cell carcinoma.\n"

"Text: {1}\n"

"Output: "

Experimental

"Gene expression is the process by which the information encoded in a gene is used to direct the assembly of a protein molecule. It's essentially how a gene gets turned into a functional product. Gene expression can be measured in a number of ways, with one common method being 'counting' the number of mRNA molecules a particular gene produces - i.e., gene expression counts.\n"

"\n"

"If a gene has a high expression count, it means that it's being actively transcribed and translated more than other genes. This usually means that the protein it codes for is in high demand within the cell or tissue. For example, genes responsible for cell growth may be highly expressed in a rapidly growing cell, while genes associated with a specific function may be highly expressed in cells that serve that function. Low gene expression means the opposite; the gene is not frequently transcribed into mRNA. It might mean that the protein product (if the gene codes for one) is not needed in large amounts within the specific cell type, tissue, or under the specific condition in which the observation is being made.\n"

"\n"

"If a gene that's typically not highly expressed suddenly shows high expression, or a gene that's typically highly expressed suddenly shows low expression, it can be a sign of certain diseases. For instance, many cancer cells have alterations in gene expression, where genes that stimulate cell division are overexpressed, leading to uncontrolled cell growth.\n"

"\n"

"Below are a list of genes that are important for predicting CHRCC or CCRCC or PRCC. The expression level of that gene in each of the three groups is given. As an example, 'TNF: low CHRCC, high CCRCC, high PRCC' means gene TNF has low expression in patients with CHRCC, high expression in patients with CCRCC, and high expression in patients with PRCC. Use the listed genes and kidney cancer expressions, given below, to help you predict CHRCC or CCRCC or PRCC:\n"

"{0}\n"

"\n"

"Output format:\n"

"{{'diagnosis': 'CHRCC' or 'CCRCC' or 'PRCC'}}\n"

"Below are gene-count pairs that represent the gene expression for a single patient with kidney cancer. Please diagnoses them with CHRCC or CCRCC or PRCC.\n"

"Text: {1}\n"

"Output: "
